# Symptomatic schwannoma of the abdominal wall: A case report and review of the literature

**DOI:** 10.3892/ol.2015.2866

**Published:** 2015-01-12

**Authors:** RUBEN BALZAROTTI, FABIO RONDELLI, JESSICA BARIZZI, ROBERTO CARTOLARI

**Affiliations:** 1 Surgical Unit, San Giovanni Hospital - Ente Ospedaliero Cantonale, Bellinzona, Ticino 6500, Switzerland; 2 Radiological Unit, San Giovanni Hospital - Ente Ospedaliero Cantonale, Bellinzona, Ticino 6500, Switzerland; 3 Surgical Pathology, Istituto Cantonale di Patologia, Locarno, Ticino 6601, Switzerland

**Keywords:** schwannoma, abdominal wall

## Abstract

Schwannoma is a rare, benign tumor that arises from the nerve sheath. This tumor usually involves the extremities, but can also be found in the head and neck, trunk, pelvis, retroperitoneum, mediastinum and gastrointestinal tract. In numerous cases, the tumors are asymptomatic and are identified incidentally on physical examination or imaging. Occasionally, schwannoma is symptomatic due to compression of surrounding large nerves. In the present study, a 57-year-old female presented to the surgical outpatient’s department due to a well-localized parietal pain in the left lower quadrant. The onset of the pain occurred three years prior to presentation, without apparent cause and in the absence of other symptoms. Ultrasound and a computed tomography scan revealed a small solid tumor in the anterior abdominal wall, which was dimensionally stable over time, but was not noted in a preliminary analysis by a radiologist. The lesion was surgically removed using an anterior surgical approach. Histopathology revealed the tumor to be benign schwannoma. The painful symptoms completely disappeared. To the best of our knowledge, this is the third case of an abdominal wall benign schwannoma in the medical literature, and the first symptomatic case.

## Introduction

Schwannoma (neurinoma or neurilemmoma) is a rare, benign tumor, composed exclusively of Schwann cells, that arises from the nerve sheath, as opposed to neurofibrome consisting of different cell types that constitute the nerve. Schwannoma is the most common tumor of the nerves and the incidence in adults is ~5% (1). It is common in neurocutaneous diseases as neurofibromatosis I; in neurofibromatosis II, schwannoma involves typically eighth nerves. It is an encapsulated lesion without an infiltrative behaviour; the lesion pushes the nerve laterally, assuming an eccentric shape, usually <3 cm in diameter. In this sense, malignant transformation is rare and growth is slow. In some locations, schwannoma can reach a considerable size, with degenerative features such as cyst, fibrosis and calcification (2). In the majority of cases, the tumor is identified incidentally and involves the limbs, trunk, head and neck. Infrequently, it can arise in any part of the body. Surgical excision is the treatment of choice. The decision whether to operate depends on the degree of the symptomatology and the location of the lesion. The balance between the relief of pain and the potential neurological deficit is a key point in the decision-making. Due to the low risk of relapse and the exceptional malignant transformation, enucleation is a surgical option (3). Partial excision can be considered in order to avoid neurological sequelae.

To the best of our knowledge, the present study is the third reported case of a schwannoma located in the abdominal wall, and the first symptomatic case (4,5). Written informed consent was obtained from the patient.

## Case Report

A 57-year-old woman presented to the surgical outpatient department due to well-localized parietal pain in the left lower quadrant. The onset of pain occurred three years prior to presentation, without apparent cause and in the absence of other symptoms. The pain was extremely localized, 6–7 cm inferiorly and laterally to the umbilicus, and was not associated with movement, position or particular events such as oral feeding and stress. Overall, the symptomatology increased over time, in terms of frequency and intensity of the abdominal pain. On physical examination, the abdomen was not painful and no mass was identified or suspected. All routine laboratory tests were normal. During the first year of symptoms, a gastroenterological outpatient consultation was performed and a colonoscopy procedure did not reveal any lesions or abnormalities. During the same consultation, an ultrasound examination was performed, notably revealing a well-defined cystic lesion, 1.6 cm in diameter, located in the muscular layer of the left-lower quadrant of the parietal wall. The nodule was not painful upon the administration of pressure. Due to the persistence of the symptoms, one year later an abdominal computed tomography (CT) scan was performed. In a preliminary analysis, no lesions were identified in the peritoneal cavity or abdominal wall, and no parietal defects were found. Three years subsequent to the onset of the symptoms, a novel analysis and review of the radiological images revealed the presence of a small parietal nodule. In order to confirm and verify the possible alteration in the tumor, a novel CT scan was performed.

**Figure 1:**
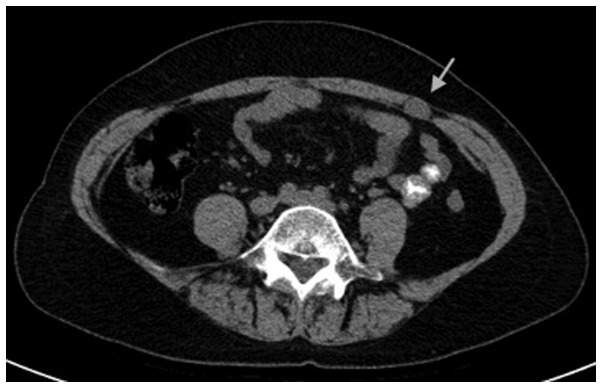
Basal computed tomography examination revealing a small, oval-shaped, omogeneous mass, with a soft tissue density that can be observed on the abdominal wall, on the left edge of the left rectus abdominis muscle. The mass possesses regular edges and is well-demarcated by fat from the sourrounding structures (white arrow).

**Figure 2:**
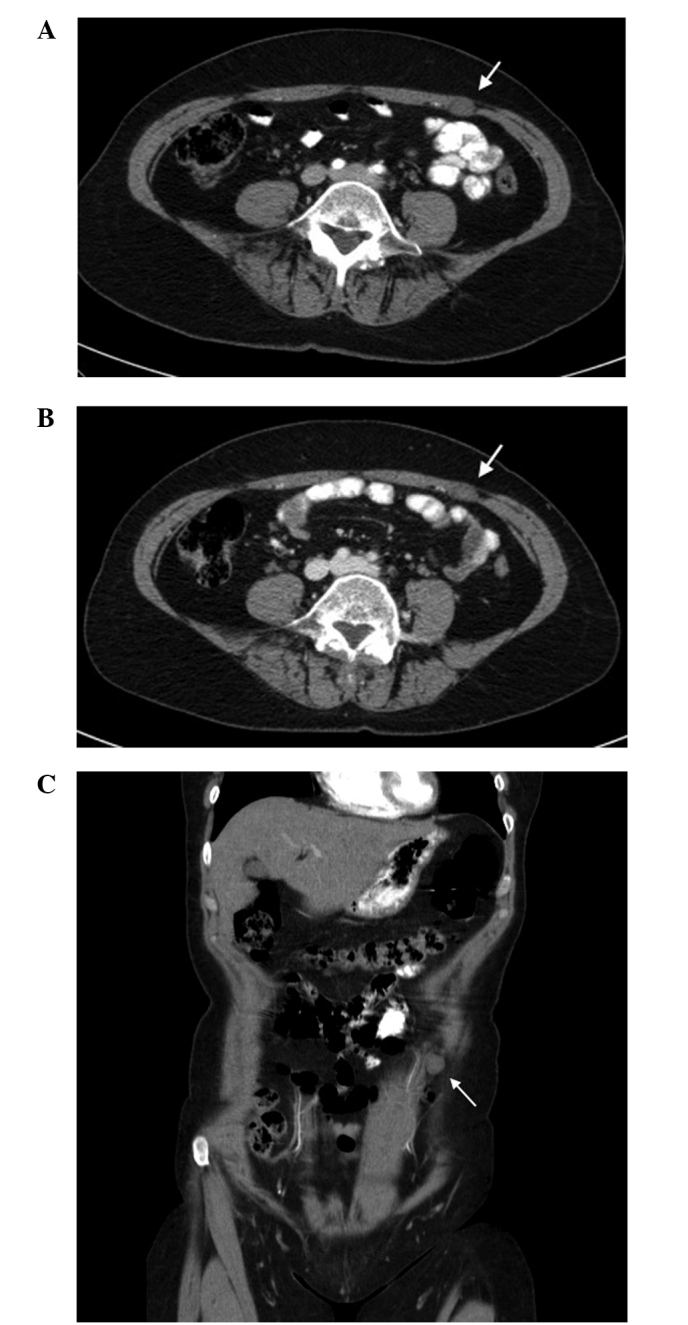
(A) Contrast enhanced-CT performed subsequent to IV administration of the contrast media. In the arterial phase, the mass exhibited a low enhancement and appeared slightly hypodense. (B) Contrast enhanced-CT performed subsequent to IV administration of the contrast media. In the venous phase, the mass remained slightly hypodense in comparison with the adjacent structures. (C) Coronal view of the contrast enhanced-CT. CT, computed tomography; IV, intravenous.

The CT scans were performed on a 16-channel multislice unit (Brilliance 16p; Philips, Amsterdam, Netherlands) and on a 256-channel multislice unit (iCT256; Philips) prior to and following the administration of intravenous (IV) contrast media with a two-year interval between the two examinations. A 17×11 mm mass was identified in the abdominal wall, on the left edge of the left rectus anterior muscle (Fig. 1). The mass exhibited a homogeneous soft mass density on basal CT, with a regular oval shape. The IV administration of contrast media for dynamic CT revealed a modest, homogeneous enhancement of the lesion (Fig. 2). No difference was identified between the first and second examinations.

The suspected diagnosis was of a benign schwannoma of the anterior abdominal wall. The most notable indication of the cause-effect association between the lesion and the parietal abdominal pain was the considerable topographic coincidence. Presented with this clear association, an indication for surgical removal was discussed with the patient and decided by mutual agreement.

**Figure 3:**
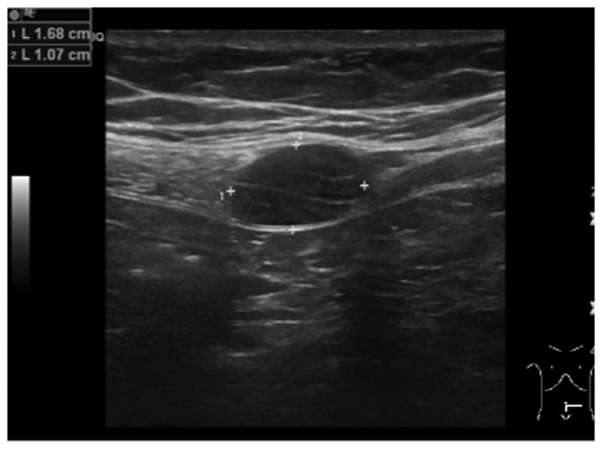
Ultrasound examination revealing a regular oval-shaped hypoechoic mass of the left abdominal wall, with internal linear hyperechoic structures, that is well-demarcated from the sourrounding structures.

A pre-operative ultrasound examination (Logiq E9 Ultrasound System; GE Healthcare Life Sciences, Chalfont, UK) confirmed the size and shape of the mass, which was hypoechoic with certain internal linear hyperechoic structures (Fig. 3).

**Figure 4:**
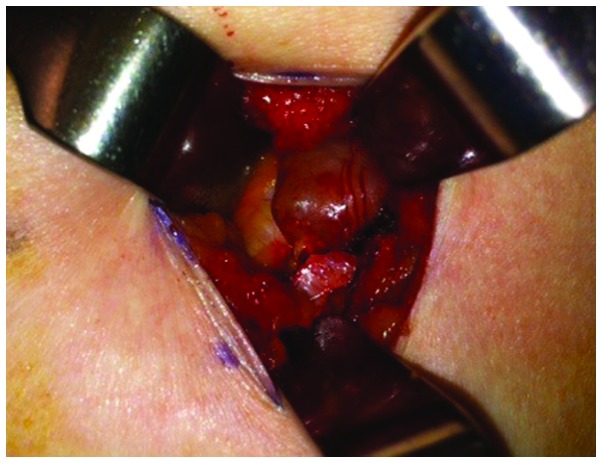
Perioperative image revealing the parietal location of the nodule, at the lateral edge of the left rectus abdominis muscle, subsequent to opening the anterior fascia.

The patient underwent surgery under general anesthesia, which was performed using a direct anterior approach. By making an incision into the anterior fascia of the left rectus abdominis muscle, the lesion was immediately identified at the lateral edge of the muscle (Fig. 4). Notably, a small parietal nerve entered and exited the nodule in an eccentric manner. The tumor was completely resected.

**Figure 5:**
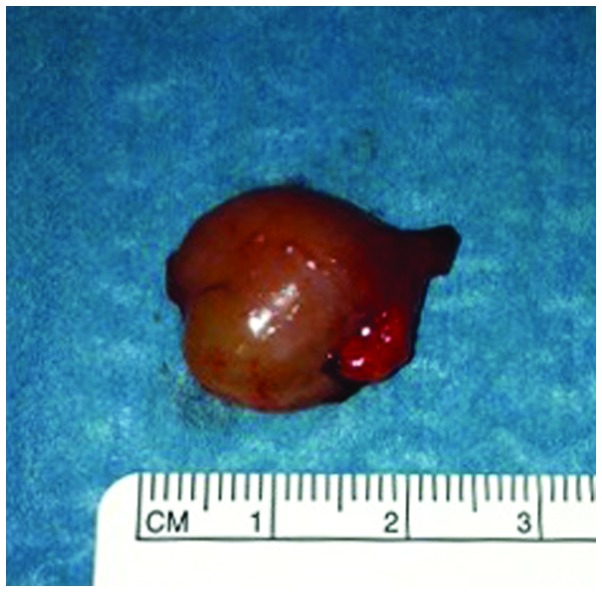
Image of the resected specimen.

Macroscopically, the nodule was 2 cm in diameter, rounded and of tense-elastic consistency, exhibiting a white color in the center of the lesion and possessing a peripheral region of myxoid appearance (Fig. 5).

**Figure 6:**
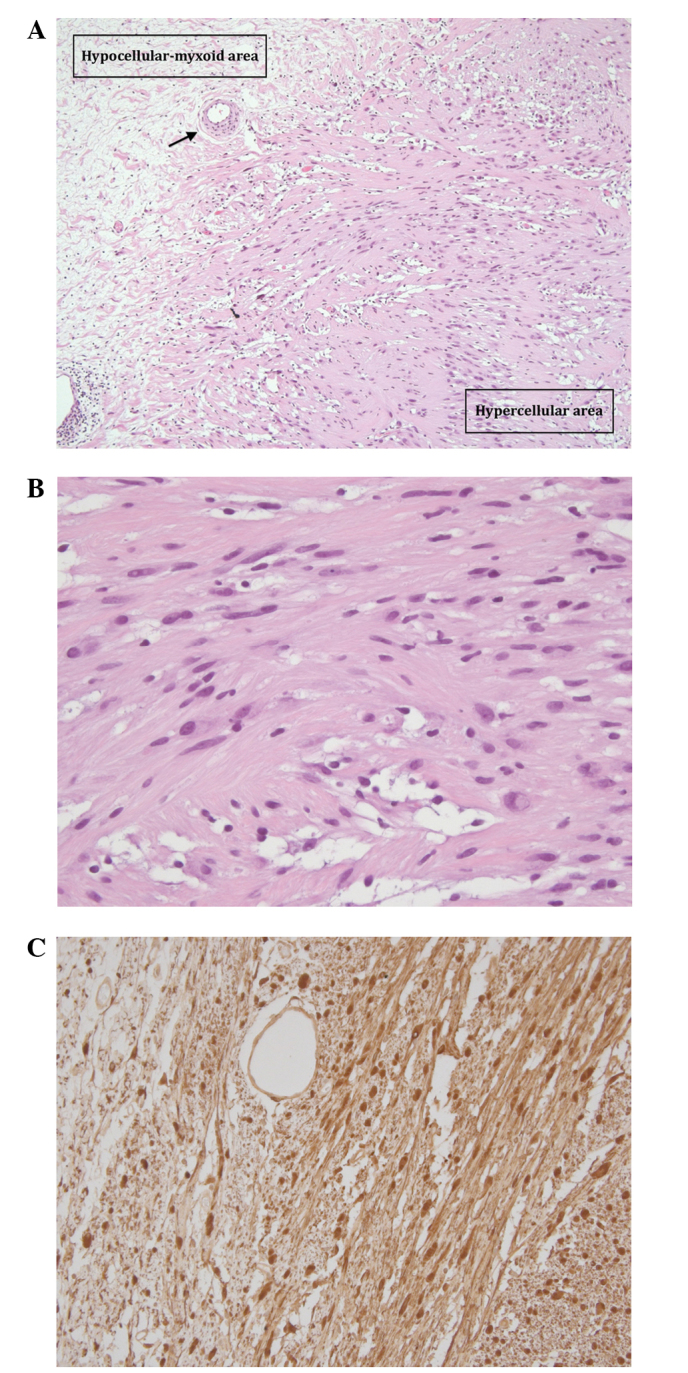
(A) Photomicrograph revealing the hypercellular and hypocellular areas. In the upper-left corner, a blood vessel with a thickened wall can be observed (black arrow). (B) Photomicrograph revealing spindle cells without significant atypia. (C) Immunohistochemistry revealing the cellular expression of the S-100 protein.

Microscopically, the lesion demonstrated an alternation of hypercellular and hypocellular areas, each comprising spindle cells without significant atypia. In the hypocellular area, foci of myxoid differentiation were also present. The tumor cells were revealed to strongly and diffusely express the S-100 protein by immunohistochemistry (Fig. 6), confirming the diagnosis of benign schwannoma. The post-operative course was uneventful and the symptoms ceased completely. A one-year follow-up appointment did not reveal any relapse of the symptoms.

## Discussion

Schwannomas are benign tumors of the Schwann cells in the neural sheath of cranial and peripheral nerves. The lesions usually involve the extremities, but can also be found in the head and neck, trunk, pelvis, retroperitoneum, mediastinum and gastrointestinal tract. However, the tumors are extremely uncommon in parenchymatous organs, such as the liver and pancreas (6). There is a female predominance and schwannoma typically occurs between the ages of 20–50 years old (7,8).

In numerous cases the schwannoma is asymptomatic and is identified incidentally upon physical examination or imaging. Occasionally, the tumors are symptomatic due to the compression of surrounding large nerves (9).

To the best of our knowledge, only two cases of benign schwannoma located in the abdominal wall have been reported in the medical literature at present. In the first case (4), a healthy 64-year-old female underwent a whole body CT scan, which revealed an incidental mass of 6 cm in the right iliac fossa. The second study (5) reported the case of a 29-year-old female who presented with a painless lump in the left-upper abdomen, which had been gradually increasing in size over 10 months. The tumor was 6 cm in diameter and, notably, the tumor was located between the rectus abdominis muscle and the lateral abdominal muscle, which is identical to the tumor location in the present patient. In the two cases, a histopathological diagnosis of benign ancient schwannoma was made.

Ancient schwannoma is a sub-type of schwannoma characterized by degenerative changes that are observed under microscopy (10). These changes are the result of long-term progression (11). In the present case, the symptomatic behavior of the lesion, which was characterized by persistent pain of the abdominal wall, may explain the early diagnosis of a schwannoma that was small in size and, accordingly, lacked degenerative changes.

As in the present patient, the identification of a nerve entering and exiting a mass is pathognomonic for a peripheral nerve sheath tumor and the eccentric association with the nerve is considered to be pathognomonic for a schwannoma rather then a neurofibroma (12,13).

To conclude, the definitive treatment for benign schwannoma is surgical removal. The prognosis of these lesions is good (14), recurrence is unusual and malignant transformation is extremely rare (15). To the best of our knowledge, the present study reports the first case of symptomatic schwannoma of the abdominal wall. A lesion located in the abdominal wall should be considered among the rare causes of unexplained abdominal pain.
